# Innovation by Computer-Aided Design/Computer-Aided Manufacturing Technology: A Look at Infection Prevention in Dental Settings

**DOI:** 10.1155/2019/6092018

**Published:** 2019-08-06

**Authors:** Livia Barenghi, Alberto Barenghi, Carlo Cadeo, Alberto Di Blasio

**Affiliations:** ^1^Integrated Orthodontic Services S.r.l., Via Camillo Benso di Cavour 52 C, 23900 Lecco, Italy; ^2^Department of Medicine and Surgery, Centro di Odontoiatria, Parma University, Via Gramsci 14, 43126 Parma, Italy

## Abstract

Recent data indicates limited awareness and compliance on infection prevention procedures by dental offices and by dental laboratories. Guidelines for infection prevention in dentistry have been published by Centres for Disease Control and Prevention since 2003; the section “IX-Special consideration” includes a subsection concerning the prevention in dental laboratories, but it has not been modernised in later versions to fit the needs of traditional and computer-aided technology. Traditional techniques required disinfecting items (impression, chewing waxes, and appliances) with well-suited products, which are also chosen for limiting impression changes or appliance deterioration. Effective procedures are available with difficulties. Some of these contain irritant or non-eco-friendly disinfectants. The transport of impression, to dental laboratories, is often delayed with limited precautions for limiting cross-infection. Gypsum casts are frequently contaminated mainly by bacteria and their antibiotic-resistant strains and even stored for long periods during dental implant supported restoration and orthodontic therapy, becoming a hidden source of infection. Nowadays, computer-aided design/computer-aided manufacturing technology seems to be an interesting way to promote both business and safety, being more comfortable for patients and more accurate than traditional technology. A further advantage is easier infection prevention since, for the most part, mainly digital impression and casts are not a source of cross-infection and the transport of contaminated items is reduced and limited to try-in stages. Nevertheless, a peculiar feature is that a digital electronic file is of course unalterable, but may be ruined by a computer virus. Additionally, the reconditioning of scanner tips is determinant for the optical characteristics and long term use of the scanner, but information for its reconditioning from producers is often limited. This study focuses on some critical points including (a) insufficient guidelines, (b) choice of proper procedure for scanner reconditioning, and (c) data protection in relation to patient privacy.

## 1. Introduction

For patient and personnel safety in dentistry, one of the main goals is to break the chain of infection transmission. Nevertheless, infection hazards in prosthodontic and orthodontic practice are rather difficult to control [1–4]. Both practices require many items: impression, models, try-in stages, and outsourced different dental appliances (customized bridges, dental implant supported restoration (DISR), different types of orthodontic appliances). In general, traditional procedures suffer from (a) compatibility problems among items and disinfectants and (b) limited compliance, errors, and lapses during infection prevention, which are very frequent in dental offices. In addition, data indicates the limited awareness of infection prevention guidelines and insufficient compliance with infection prevention by most dental laboratories (DLs) during the manufacture of dental prostheses and orthodontic appliances [5–10]. Contaminated items often come and go from the clinic to the DLs and vice versa, and this increases the hazard, the possibility of microbial reservoirs, and the chance of infection transmission [2, 5–13].

The limited compliance with infection prevention is hazardous, taking into account the increasing prevalence of infections by antibiotic-resistant bacteria, killer bugs, or super spreaders [14], and the growing number of elder dental patients with impaired immune defence system; frequently, they need complex and cutting edge surgical procedures and prosthodontic treatments, which unfortunately also have been associated with incidents of malpractice [15, 16].

Nowadays, computer-aided design/computer-aided manufacturing (CAD/CAM), here indicated as CAD/CAM technology (CCT), is widely used since (a) digital impression is more comfortable for patients (mainly vulnerable aging population or younger ones) [23]; (b) accuracy of prosthetic restorations is equal or higher than conventional restorations [24]; (c) it significantly reduces the costs (about 30% per each crown) and the active working time (90% for final crown) [24, 25]; (d) the exclusive use of aesthetic and more biocompatible materials (i.e., zirconium oxide, lithium disilicate) [15, 26, 26]; (e) the flexibility to manufacture from simple crown to complex DISRs and orthodontic appliances [25–31]; (f) the appeal of virtual technology to promote business; and (g) it improves ecofriendly dentistry [3, 32]. A recent survey shows that restorations by CCT represent a significant innovation adopted by dentists in New Zealand and have been greatly appreciated by dental students [33, 34]. The global dental CAD/CAM & dental prosthesis market is increasing significantly: it was estimated at about 9,400 Mn USD by 2018 growing at a compound annual growth rate of 5.6% over 2024 [35].

Despite the high interest in dentistry on CCT nowadays, gold standard guidelines for infection prevention did not pay attention to it [1, 36]. Concurrently, insufficient notes are available from Laws on health safety and guidelines [37].

Using CCT, the usually reported advantages for infection prevention are the none requirements of impression disinfection and better occupational safety. To our knowledge, this is the first paper that focuses on infection prevention in detail using CCT compared to traditional technology in dentistry. Here, we report three specific problems related to (a) insufficient guidelines, (b) requirements for scanner reconditioning, and (c) data protection and electronic viruses.

## 2. Materials and Methods

### 2.1. Information Sources and Search Strategy

The electronic literature search was conducted via the PubMed and Google Scholar databases (from January 2010 up to and including October 2018) using various combinations of the following key indexing terms: (a) CAD/CAM; (b) cross-infection control; (c) infection prevention; (d) disinfection; (e) reconditioning; (f) semicritical items; (g) critical items; (h) cast; (i) digital model; (j) digital impression scanner; (k) dental impression; (l) guidelines; (m) safety precautions; (n) dental laboratory; (o) occupational health; (p) bacterial adhesion; (q) microbial contamination; and (r) biofilm. In addition, manual searches were carried out in the Hindawi Journal database (from 2010 to 2018) using the following key indexing terms: (a) CAD/CAM (n°=237); (b) CAD/CAM dentistry (n°=118), but very few take into account cross-infection or infection prevention according to our topic [38, 39]. Subsequently, bibliographic material from the papers has been used in order to find other or older appropriate sources in relation to specific topics and operative problems. A total of 108 papers and links were found suitable for inclusion in this paper. Only a few papers do not have a DOI or PubMed classification, but the available Internet link and date accessed have been added.

## 3. Results and Discussion

### 3.1. Background

Currently, there is increasing interest regarding safety of the dental workplace, personnel, and patients and in particular, on the prevention of infectious adverse events and clinical hazards. Adverse events and outbreaks mainly cause an increase in the cost to society (by productivity loss, additional costs for health care, outbreak investigations by molecular diagnostics), and significant legal claims [40, 41]. In addition, a burning issue is the growing number of susceptible patients (HIV positive, diabetic, the elderly, those under frequent antibiotic treatments or chemotherapy, women in pregnancy, children, teenagers) with an impaired or underdeveloped immune defence system; in addition, other patients show oral lesions or tissue trauma after clinical treatments (i.e., preparation of the cast crown, impression, trying practice of orthodontic band selection, etc.) or gingival inflammation. In all these cases, the chances of infection are expected to increase.

On the whole, dental impressions and appliances from all persons must always be treated as if potentially infectious (by microbes present in saliva, occult blood, dental plaque), since persons could be in an asymptomatic stage (early stage of Hepatitis C infection) and could not know their status, or the infection may be diagnosed late, or undeclared to avoid discrimination (HIV infection). Furthermore, the recommendation to isolate prosthesis of high-risk patients from other laboratory work in dental offices and DLs is nowadays without a rational reason and dated.

Moreover, we have to make every effort to reduce the rate of infection transmission to/from dental offices and to/from laboratories and the chance of there being some microbial reservoirs (impression, dental appliances, etc.). Conventional fabrication methods require considerable human intervention and manipulation of impression, wax and cast, materials and try-in-stage items; as a consequence of these two peculiar features, items exhibit microbial contamination caused by the bioburden of the oral cavity, hand skin, environment, and even by some harmful antibiotic-resistant strains. Here, we focus on some underestimated hazards and operative errors and lapses during infection prevention using traditional technology and CCT.

### 3.2. Failures in Infection Prevention in Dental Offices Using Traditional Technology

More recent findings indicate insufficient knowledge and very limited awareness by dental healthcare personnel (DHCP) in relation to infection control, taking into account the insufficient use of PPEs, low use of sterilized impression tray (13%), rinsing the impressions with water (37.2%) or brushing away debris (2,6%) before disinfection, blood-contaminated impression (25%), improper disinfection of impression (about 40%) or of metallic impression trays, denture prosthesis, bite registration and wax, face bole and fork, and lack of communication (24,7%) with DL about impression disinfection in dental offices [2, 11–13, 39, 42–46].

**Figure 1:**
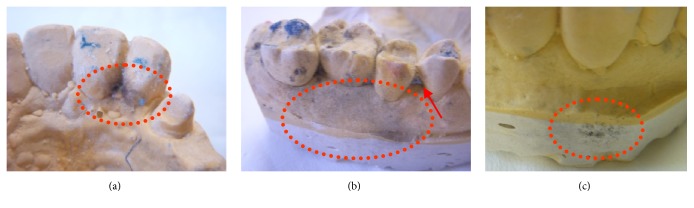
Some examples of cast microbial contamination (see bluish-black hairy colonies probably from Fungus species) due concurrent factors (improper impression disinfection, prolonged conservation inside the plastic bag, presence of alginate residues after manufacture steps in a traditional DL). The casts represent a hazardous reservoir since DHCP hand-touching.

In brief, many opportunistic or nonopportunistic species (i.e.,* Staphylococcus*, Methicillin-resistant* Staphylococcus aureus *(MRSA),* Candida, Pseudomonas, Enterobacter cloacae, Escherichia coli*,* Klebsiella oxytoca, *Streptococcus, Actinomyces, Enterobacter,* Klebsiella pneumonia*) have been reported on impressions, dentures, crowns, and wax occlusion rims [47, 48] (Figure 1). Sofou's group reported that seventy-two percent of the impressions were contaminated at a low level (median number of 1.3 × 10^2^ cfu/20 mm^3^), while thirteen per cent of the samples yielded at a higher range (10^3^- 3.4 × 10^4^ cfu/20 mm^3^) [49]. Most of the isolates were non- or *α*-haemolytic bacteria and presumably low-pathogenic strains. Nevertheless, we would like to note that* Bacillus *strains, mainly nonpathogenic species and well adapted to the human host, have been reported to contribute to latent infections and/or to reactivate others (i.e., HIV, tuberculosis) [50]. In addition, since they are spore forming species, they are difficult to eradicate from stone casts [49–51]. More recently, bacterial contamination, checked by using molecular biology techniques, was also found on the final removable orthodontic appliances (~10^2^–10^3^ cfu/ml); nevertheless, the contamination derives from the DL rather than from patient's impressions [52].

MRSA is a well-known antibiotic-resistant bacterium with a very low expected infective dose (4 CFU) [14]. Impression material cartridges and handgun dispensers are easily and heavily contaminated with pathogenic agents, including MRSA, during clinical prosthetic procedures [53]. The infective hazard is expected since most invasive dental procedures are performed in dental surgery and prosthetic wards, where patient bleeding is frequent. Nowadays, the use of heat-sterilized hand-pieces and proper water quality during prosthetic tooth preparation, because of the frequent bleeding, is absolutely necessary [1, 36, 54]. On clinical contact surfaces, the MRSA contamination was higher in samples from dental surgery (4.3%) and prosthetic dentistry (3.9%) compared to prosthodontic procedures (1%) that are mostly none invasive [55]. Nevertheless, it is hazardous that the majority of MRSA and* Staphylococcus aureus *isolates, recovered from environmental surfaces, were biofilm producers [14, 22, 55]. The contamination of MRSA is high in conventional impression and gypsum casts: it has been found in 15.4% and 27% of them, respectively [48, 56].

We would like to note some underestimated hazards during traditional prosthetic, prosthodontic, and orthodontic practices as follows:

- Concerning the procedure using an addition silicone as impression material, the contamination by the hand microbial flora (including MRSA) [14, 22] is expected during the mixing of the base and its catalyst with ungloved hands. Recently, this problem can be avoided using powder free latex gloves or impression material automatic mixers.
- It is a frequent error to touch a cast or contaminated try-in items with gloved contaminated hands [4]. Therefore, it is not a surprise that casts are frequently contaminated mainly by bacteria and MRSA and could be a source of infection [4, 22, 56]. We would like to note that contaminated casts come and go between the dental office and DL and/or are utilized for long periods during DISRs and orthodontic cares; then, they are a hidden source of contamination (Figure 1). Nothing is known about the contamination of articulators, but it is expected to be high.
- During partial- or full-denture impressions, it is a frequent error to use the* big *brush of the rubber base adhesive without disinfecting the appliances or the customized tray. We should note that the isolated species from the denture surface are aerobic bacteria, fungi, Enteric rods,* Candida* spp.,* Pseudomonas* spp.; they are generally part of the normal oral flora, but could be pathogens for immune-compromised patients, while anaerobic species should colonize the internal porous system of the acrylic resin of the removable appliances [12, 13]. Then, the contamination of the brush and the adhesive are expected, but this should be inconsistent with recognized standards of infection control.
- The transport of contaminated impressions, chewing wax, and intermediate tests during prostheses are often carried out without proper precautions against cross-infection, with violation of the national laws, as well as being delayed. Concerning the disinfection of traditional impressions, the instructions for use (IFU) from manufacturers are often insufficient or not usable (i.e., very restrictive use of glutaraldehyde in European Union). The responsibility for ensuring impressions has been cleaned and disinfected before dispatch to the laboratory which lies solely with the dentist.
- When at the dental chair, the modification of removable orthodontic and prosthetic appliances should be avoided before try-in and after use by all patients, without their preliminary disinfection. We would like to note that removable prosthodontic appliances received from laboratories are often contaminated by* Bacillus* spp. (57% of the isolates),* Pseudomonads* (22%),* Staphylococci *(13%), and* Candida* species (38%). In addition, acrylic base plates are always contaminated by* Streptococcus* biofilm even after short usage [4, 13]. An option for avoiding the environmental contamination and occupational hazards is to modify appliances inside a closed equipment (usually called dental sandblasting equipment) with dust aspiration.

Concerning specific problems on impression disinfection, we add additional operative details in Section 3.4.

### 3.3. Contamination of Dental Impression Materials from Manufacturers

Insufficient data exists on the contamination of dental impression materials supplied by the manufacturers in sealed containers. They are mainly stored in an anhydrous state and are often hydrophobic, which means the microbial contamination is expectedly low. Extra-mouth contaminants represent only 0.06% of total microbial (aerobic mesophilic bacteria) load of alginate, while after mouth contact, alginate microbial load increases significantly (1600 fold); other powders, from impression material containers and irreversible hydrocolloids received from the manufacturers, were contaminated with viable microorganisms to a substantial amount (90-100% in irreversible hydrocolloids) [57]. It is unclear if dental impression materials themselves can act as vehicles for microorganism transmission or be a hazard for immune-compromised patients [58].

### 3.4. Focus on Impression and Cast Disinfection Using Traditional Technology

Firstly, the use of all PPEs is always required because of the infective risk and the occupational hazard due to splash in the case of immersion, air contamination in the case of spray, or dryout with compressed air. Studies, among DHCP and dental technicians within different Nations (UK, Pakistan, South Arabia, Iran), indicated a wide variety of chemical solutions and concentrations were used to disinfect impression materials [10, 44, 45, 59–61].This is indicative of the degree of confusion and difficulties in the choice of the proper disinfectant with inadequate recommendations and insufficient knowledge. Data mainly focuses on the effects of disinfectants on impression surface details and dimensional accuracy of items (impressions, master casts, etc.) caused by different reasons [62, 63]. Taking into account the conditions encountered in clinical practice, unfortunately, data is lacking on the effects of procedure delay [64]. Frequently, an alginate impression is placed in plastic bags with moist cotton, but the delayed delivery to the DL of inadequately disinfected impressions could allow for microbial growth during storage. Using conventional technology, it is very important to firstly remove blood and saliva contamination that can alter bacterial adherence capacity, while it is not clear to what extent (0-90%) the preprocedural rinsing of the impression with tap water should significantly remove bacteria and increase the efficacy of subsequent disinfection [5, 49, 57, 65, 66].

In general, the impression disinfection, in a dedicated area near the chair side area, is an ideal way to prevent cross-contamination. Many studies report impression surface disinfection with different commercial products, by spay or immersion and with a contact time of about 5-10 min. Disinfection by soaking in chemical materials has been shown to cover all surfaces of impression materials at one time, while spraying is not capable of disinfecting all surfaces effectively and also cannot cover all undercuts.

It is preferable to avoid the use of irritants (aldehydes, hypochlorite solutions), or non-eco-friendly disinfectants (aldehydes, phenols). Hypochlorite solutions, very effective and cheap products with no or minimal certification, may have corrosive or discoloration effect on prosthesis metal parts as far as occupational hazards [67]. The safer disinfectants specific to this area are based on alcohols, chlorine combination, chlorhexidine ± enzymes, biguanides, and ammonium compounds.

Recently, more ecological approaches have been proposed for dental stone and impression disinfection using microwave and UV radiation [51, 68, 69]; these procedures should avoid the possibility of surface deterioration as they do not involve immersion/spraying of the impression with disinfectant.

### 3.5. Impression Tray

**Figure 2:**
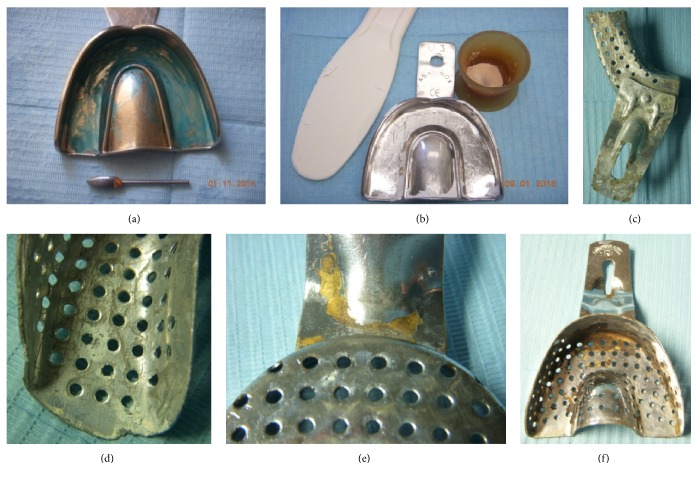
Residues of polyether adhesive, alginate, and autopolymerising acrylic resin on impression trays and other items (alginate spatula, laboratory bur, silicone dappen dish) after treatment by washer-disinfectors (a, b) and corrosion of impression trays by an improper or overly long chemical treatment to remove gypsum (c-f). Tips: (i) remove residues before treatment by washer-disinfectors (a, b) [17]; (ii) these impression trays must be promptly substituted (c-f).

Before further reconditioning [36, 70], patients' reusable impression trays must be perfectly cleaned of bioburden and of residues of adhesive and impression materials, cements, adhesive, and gypsum, using self-acting products. It is well known that the prolonged immersion of metal trays using specific products may cause some corrosion, mainly of aluminium or chromate trays (Figures 2(c)–2(f)). Careful attention should be given to hazard identification and precautionary statements (indicated in MSDS) of cleaners for alginate and gypsum residues. The preliminary removal of any residues from impression trays is needed since further mechanical action by ultrasonic devices or washer-disinfectors is not able to remove them and would impair further disinfection and sterilization (Figures 2(a) and 2(b)).

### 3.6. Failures in Infection Prevention in DLs Using Traditional Technology

When traditional technology is used, the work in DLs comes with many physical, chemical, ergonomic, and biological hazards [6–8, 71]. Despite the lack of contact with patients, there are many opportunities for cross-contamination throughout the manufacture of the appliances.

Data shows the limited awareness on infection prevention and very poor compliance of infection control procedures by most DLs during the manufacture of dental prostheses and orthodontic appliances; in particular, studies show inadequate adoption of standard precautions in terms of the use of PPEs, disinfection of impression and appliances, and vaccinations [5–10]. DTs are exposed to microorganisms via direct contact with nondisinfected items (i.e., impressions) through cuts and abrasions mainly on ungloved hands. A recent study reported that DTs received 95% of blood-contaminated impressions and 15% had encountered blood-filled voids upon trimming back the peripheries of impressions [11]. The risk of cross-infection between the clinical and DL settings seems to be significant; when during '90 yrs, DT adopted very limited infection prevention procedures, and they showed significantly higher exposure to HBV than a comparable population (2.7% vs. 0.76%) [72]. Despite this hazard, the percentage of vaccinated technicians against HBV is unsatisfactory, ranging from 10 to 60% [7, 59].

It is well known that the storage plus transport of impression to DTs takes from 5 to 8 hrs in moist conditions; the influence of humidity on microbial survival is a recently discovered problem; for example, HBV can survive for up to seven days in 42 percent relative humidity. A survey shows that 50% of the responding DTs disinfected all impression partly from uncertainty (no written communication) or inefficiency of disinfection in dental offices [11]. Nevertheless, repeated disinfection has been reported to influence surface detail and the accuracy of the impression. Chorexidine, a disinfectant often used prior to final packaging and dispatch of the custom-made appliances, has been reported to deteriorate the acrylic surface of appliances [5] and recently involved in antibiotic resistance. Furthermore, chemical disinfectants affect the physical properties of the gypsum materials when used as water mixing substitutes; this approach has therefore been discarded by manufacturers [73]. However, the gypsum-based stone model preparation by an exothermic setting reaction may reduce the viable bacterial content on the impression as well as the cast.

In addition, other factors could jeopardize infection prevention: the need to rush a case, the absence of disinfection areas within their dental laboratories, and low awareness of legal responsibility towards occupational risks [5–10].

Only 6.40% of DTs use all PPEs and just 45.6% stated that they clean and disinfect their work surfaces. Astonishingly, 47,8% of DTs only cleaned the rag wheels, brushes, and acrylic burs with water after use, and only 28.26% of them sterilized by heat or chemicals [59]. When polymerizing, grinding, or polishing, the chance of cross-infection is still severe due to heavily contaminated dental pumice, slurry, the brushes, and heated water baths [74–77].

These worrying practices render the rest of the precautions useless because infective agents are able to survive on contact surfaces, air, hand, and work items for several days and then could contaminate already disinfected appliances. Recently, DT behaviour seems to have got worsened as concluded by some authors. Vasquez-Rodriguez's group concluded: ”*Substandard cross-contamination practices seem to be a common finding in dental laboratories, which may well compromise the quality of certain dental treatments*” [10], while Diaconu noticed that the majority of technicians were aware of the existence of a real contamination risk, both of the lab surfaces and the personnel; however, the economic crisis has forced them to reduce the lab budget for infection prevention, and vigilance [78].

### 3.7. Regulation and Recommendations

The recent European Union Regulation n° 745/2017 reported only a vague indication to health safety and some notes on cleanliness and sterility of dental appliances, all classified as medical devices (MDs), placed on the market. There is no specific guidance issued to dental custom-made MDs in contrast with the fact that dental appliances should be free of microbial contamination according to CDC guidelines [1, 36, 37]. The guidelines for dentistry published by CDC* since 2003,* include, as Special Consideration, a subsection called Dental Laboratory, but it has not been updated in later versions [1, 36]. In addition, guideline recommendations or other requirements should reflect what the field regards as good practice, but, in this case, updated instructions from the FDA and Dental Federations (International Dental Federation, American Dental Association) are insufficient [79] or refer to CDC previous guidelines* set in 1993* [70, 80, 81]. CDC guidelines for implementation suggested to “*Consult with manufacturers regarding the stability of specific materials (*e.g.,* impression materials) relative to disinfection procedures' including specific information regarding disinfection techniques used (*e.g.,* solution used and duration), when laboratory cases are sent offsite and on their return*” [36].

Up to now, there are no disinfection protocols which have been accepted as gold standard for disinfecting dental impressions and dental appliances. Chemical disinfection is still the method of choice since sterilization with heat is not an option for dental impressions and occlusal records.

### 3.8. Infection Prevention Using CCT Compared to Traditional Technology

The dental service market is always becoming more competitive. Today, the increased ergonomics of the highly complex “human-technical dental office system” are very important in guaranteeing safer and patient-centred dental care concurrent with earning profit [14]. Apart from clinical advantages and limitations already discussed by many authors [6, 8, 10, 15, 29–31, 71, 72, 78, 82–86], CCT seems to be a promising way to prevent cross-infection.

Here, we show the main differences in the case of traditional vs. CCT, mainly focusing on dental offices (Table 1). The biohazard for dental patients and DHCP is greatly reduced using CCT largely due to reduced contamination during digital impression in the dental offices and further digital manufacturing of appliances in closed automated conditions (printing technologies for polymer and metals; metal, zirconia, ceramic, PMMA milling technologies) with mainly environmental contamination. The most modern production process is fully automated and milling machines are equipped with automatic systems for the replacement of tools: this allows, starting from the raw materials, the possibility of arriving at finished dental appliances with limited or without human intervention. The residual biohazard should be prevented by the use of PPEs and adequate infection prevention during the service of rotary cutters, filters, and internal parts of the milling machine, etc. Finally, quality control and appliance disinfection before delivery are easier and automated using CAD/CAM compared to traditional DLs. More attention is needed during the handling of try-in cases and ready dental appliances. These appliances, considered semicritical items, should ideally be sterilized or receive at least intermediate-level disinfection (tuberculocidal claim) before the delivery in a sealed pouch to dental offices.

Other advantages are related to the following:

- Better occupational safety for DHCP and DTs by avoiding [87]:
  - Skin irritation after extensive use of disinfectants for impressions and dental waxes.
  - Silicosis by exposure to airborne particles liberated during the mixing of alginate (dust, lead) in dental offices and melting, grinding, polishing, and finishing procedures in labs.
  - Nonexistent or low biohazard due to waste management.
  - Higher hazard for the younger DHCP, who are concurrently more exposed to cross-infection, mainly when they are students and in the first years of their dental practice [34, 42, 88].
- Progress towards ecofriendly dentistry by reduction of
  - disinfectant use
  - waste material (contaminated gypsum and cast) [32]
- Clinical biosafety because
  - the violation of critical anatomical features is prevented by marginal fit lower than the clinically acceptable value [85]. In particular, the accuracy of DISRs by CCT is determinant in order to avoid microbial niches between prosthesis and connecting elements (implant abutment) [86, 89].
  - the new dental materials (i.e., PMMA, zirconia), usable only by CCT, show reduced adhesion and decreased biofilm accumulation [90, 91].
  - DHCP can minimize the risk of osteonecrosis, a rare and unexpected complication during the taking of conventional dental impressions in patients with predisposed anatomic sites, or the risk of* Candida* transmission in patients with denture stomatitis, a very common condition found among the elderly population [92, 93].

CCT disadvantages are on the prohibition of use on patients with pacemaker and minor occupational hazards (eye safety, extended computer usage, and ultrafine particles and nanosized byproducts) [6, 8, 10, 75, 94].

### 3.9. Factors Influencing Intraoral Scanning for Digital Imaging

The scanner is a very responsive appliance. Several factors have been reported to influence the accuracy of the intraoral scanning including (a) the learning curve, skills, and scanner usage frequencies in clinical practice; (b) the physical resolution of the scanning system and the postprocessing of the data; (c) the movement of the patient and limited intraoral space; and (d) temperature fluctuation; (e) the presence of moisture, water, saliva and sulcular fluid, and reflective surfaces (metal brackets and implant abutments) [95–101]. It is not known if the presence of* occult* blood in saliva or sulcular fluids or some of their compounds (perhaps hemoglobin, lactoferrin, volatile compounds, glandular mucous) may influence the direct scanning of a tooth prepared subgingivally, for example, or an abutment coupling. In general, scanning technology has to improve (a) the speed of the scanning process (with both hardware and software improvements), (b) the size of the scanner wand and the design of a thinner scanning tip to improve patient comfort, (c) proper devices for a better dry field, and (d) increased resistance to reconditioning and sterilization of the apparatus.

When the powdering procedure is needed to prevent reflections during image capture, there was no way to standardize it for each scan, it is not appreciated by the patient, and the environmental contamination caused by titanium powder nanoparticles is not known.

#### 3.9.1. Unit Hygiene and Scanner Tip Reconditioning

In line with the current minimal requirements for the indication of hazards published [81], the importance and the problems derived from scanner tip reconditioning have not been taken into consideration by other authors [24, 82, 83, 96–98]. We evaluated IFUs indicated for two scanners by TRIOS® and iTero® [19–21] (Table 2). Only iTero® recommends different cleaning and disinfectant commercial products for use for the Scanning Unit and the Base Unit; these disinfectants are often a mixture of different disinfectants (alcohols, Quats plus alcohol, Hydrogen Peroxide), fast acting and with a broad spectrum of activity, and all have clear certifications according to regulations. TRIOS® is rather confusing on IFUs found in two different manuals [19, 20]. IFU mainly contains recommendations for using disinfectants (60-70% alcohol-based ones) to prevent mirror damage and strict prohibition on other types of disinfectants to clean the tip mirror (i.e., ammonia-based or chloride based solutions, acetone, any oxidizing solutions) indicated in the online manual [20]; nevertheless, another IFU allows high-chemical disinfection with Wavicide®-01 and Cidex OPA® solution, if allowed by National rules. To our knowledge, it is a bizarre indication since aldehydes should be avoided on other optical devices (dental curing light) because of their ability to precipitate on optical fiber [102]. TRIOS® does not indicate potential explosion hazards if in the presence of residual flammable products (i.e., alcohol-based disinfectant), except inflammable anaesthetics. Conversely, iTero® uses disposable plastic sleeves for patient scanning. High-level disinfection was possible for 50 and 150 cycles, respectively, for tips with TRIOS® scanner tips with fixed mirror and detectable mirror, while for Carestream®CS 3600 up to 20 cycles [103]. In general, scanner producers always underline not touching the optical surface with gloves, while there are no indications for avoiding the use of powered gloves during reconditioning and the replacement of disposable sleeves.

#### 3.9.2. Some Advices

Finally, we would like to underline some advice for avoiding (a) lint, stains, and dirt on the optical components, (b) damage on optical component, and (c) fast deterioration of the plastic parts of the unit (Table 3).

### 3.10. Retraction Cord

It is well known that gingival retraction procedures are part of impression procedures; generally, this step is considered “safe” and effective, but also time-consuming, uncomfortable for dental patients, and may delay periodontal tissue repair [104]. The retraction cord is needed, also in the case of CCT, since the difficulty in scanning subgingival margins (>1 mm); in this case, dry retraction cord is used. When wet retraction cord is used during a traditional procedure, retraction cord contamination is expected. In fact, a very frequent lapse is to wet the retraction cord into the solution of topical haemostatic agents (sold in very little bottle, but that is used for long periods) without cross-infection precautions (i.e., use of unsterile College plier).

### 3.11. Data Protection and Infection Prevention from Computer Viruses Using CCT

Data protection is at the core of the recent EU General Data protection Regulation [105].

All dentists and DTs must pay attention to the health data of their patients, in terms of purpose limitation, data minimization, accuracy, storage limitation, integrity, and confidentiality. Data retention and reuse time must be explicit, while the need to retain the files for defensive dentistry (i.e., medical-legal and insurance reasons) or for future appliance repair is a matter of discussion. Orthodontists can easily backup the digital data and keep them for at least 10 years; meanwhile, gypsum casts could be lost or broken or need space in dental office, in addition to being a hidden source of contamination [106, 107].

The main advantage of CCT depends on the capability of forwarding some images, static or dynamic, coming from different sources (digital camera, CBCT, video, etc.), to a milling centre that will integrate them using Digital Smile Design software. After elaboration and dentist approval, the files will be used by computer-aided design (CAM) software to guide robotic devices which create objects and eventually assemble their parts in a virtual environment. Concerning the safety of the digital workflow, it is highly important to stay protected by installing a robust antivirus program, to protect key functions, applications, and emails and mainly to prevent the copy/deletion/stealing or encryption of the patient's personal and sensitive data. It is obvious that digital dental casts can be controlled more easily by computer cryptographic and pseudonymisation tools, than by paper documents and analogue casts; this feature is expected to prevent clerical errors, involved in the majority of patient safety incidents [18].

## 4. Conclusion

In every day practice, CCT is one of the most important innovations that support infection prevention compared to traditional technology since it breaks or reduces cross-infection during impression and manufacturing steps. These advantages are expected to balance the higher cost of investments in hardware (scanner in the dental office and CAM in the milling service and dental labs) and software for “digital smiles”.

As life expectancy increases, the prevalence of Alzheimer's disease will increase even further. Dentistry seems to be in the first line of prevention and should begin to equip itself with skills, updated knowledge for taking care of the different needs, and demands and aspirations of typically aged and Alzheimer's patients, including innovation through digital dentistry [108].

Unfortunately, guidelines for infection prevention using CCT have not been updated. DHCP needs better IFU and transparency from manufactures. Additionally, the presence of an infection prevention coordinator is necessary to follow IFU, as well as a plan for coordinated infection prevention between dental office, DT, and milling centre.

It is necessary to respect patients' rights in terms of privacy and large data protection.

## Figures and Tables

**Table 1:** Main differences for cross-infection prevention in the case of traditional technology vs. CCT in dental office and DL.

n°	Need for	Traditional Technology	CCT
1	effective communication and coordination between the dental office and laboratory efforts to asepsis	yes	only in the case of intermediate and completed cases

2	written information regarding the methods (e.g., type of disinfectant and exposure time) used to clean and disinfect the material (e.g., impression, stone model, or appliance) and items (articulators, case pans, or lathes) according to the manufacturer's instructions.	during all phases	only in the case of intermediate and completed cases

3	heat-tolerant items used in the mouth (e.g., metal impression tray or face bow fork) that should be heat-sterilized before being used on another patient or single-use plastic impression trays	yes	only for scanner tips

4	clean and disinfected pressure pots and water baths between patients since these are particularly susceptible to contamination by microorganisms	yes	No/ only for positioning wax

5	wearing appropriate PPE (including eyewear!) in both the office or laboratory, when handling contaminated items and until disinfection is completed	yes	only in intermediate and completed cases and after the end of the CAD

6	guarantee that the appropriate and effective cleaning and disinfection procedures are performed in the dental office or laboratory	+++	+

7	use an EPA-registered hospital disinfectant with a tuberculocidal claim, follow IFU and thoroughly rinse item before being handled in the in-office laboratory or sent to an off-site laboratory	yes	no

8	checking IFU and problems regarding the stability of impression and appliance materials during disinfection	yes	no

9	cleaning and disinfection of any items (impressions, prostheses, or appliances) as soon as possible after removal from the patient's mouth before drying of blood or other bioburden that can occur	yes	only in intermediate and completed cases

10	a separate disinfecting, sending, and receiving area should be established to reduce cross-contamination in the dental office	yes	easier and only in intermediate and completed cases

11	identification and reduction of redundancies of procedures since impression materials could be damaged or distorted because of disinfectant overexposure	yes	no

12	cleaning, disinfecting, and covering of clinical contact surfaces as a function of the rate of use and contamination of the area	+++	+

13	fabricating stone casts after alginate impression as soon as possible to avoid dimensional changes	yes	no

14	adhesive for impression trays using some impression materials (polyether, polysulfide)	yes	no [18]

15	wastage of impression materials due to the remaking at times of conventional dental impression for inadequate detail production	yes	no

16	wastage of time due to the remaking of dental impression for inadequate detail production	+++	+

17	appliances and prostheses that should be free of contamination delivered to the patient	difficult	easy

18	responsible dental laboratory or dental office staff for the final disinfection process	yes	yes

19	a separate receiving and disinfecting area should be established to reduce contamination in the DL	yes	in intermediate and completed cases

20	waste (gypsum, waxes) management according to national laws	yes	no

21	Appropriated disposal of gypsum and toxic substances (i.e., hydrogen sulphide) when discarded into the environment	yes	no

22	laboratory items (e.g., burs, polishing points, rag wheels, or laboratory knives) which are heat-sterilized, disinfected between patients, or disposable items, or to store items in small quantities (i.e., polishing agents)	yes	low and only to reduce manufacture contamination

23	regulated medical waste and sharp items (e.g., burs, disposable blades, and orthodontic wires) in specific and resistant containers according to national rules	+++	+

24	paper for dentist prescription to DL	yes	no

25	computer antivirus	no	yes

**Table 2:** IFU according to infection prevention from different manufacturers of scanners [19–21].

	Specific Indications	
Part of the scanner	TRIOS®	iTero®
System or Base Unit [20]	(i) Surface disinfection	(i) Surface disinfection.

Monitor [20]	(i) Do not spray directly with disinfectant.	(i) Do not spray directly with disinfectant. (ii) Use disinfectant wipes for the Scanning Unit and Base Unit.

Handheld scanner [20]	(i) Do not submerge the handheld scanner in any liquids. (ii) Do not place the handheld scanner on heated or wet surfaces. (iii) Surface disinfection.	Not indicated in open source [21].

Medical-grade peripherals (e.g., keyboards and mice) [20]	(i) Easy disinfection.	Not indicated in open source [21].

Scanner tips with fixed mirror or detachable mirror [19]		Not needed.
	Immediately after clinical use: (i) Detach the mirror from detachable mirror and go on reconditioning separately for tip and mirror. (ii) Go on reconditioning for tip with fixed mirror.	
	(iii) Clean manually and perfectly using soapy water and a soft dish brush. (iv) Rinse carefully the tip. (v) Inspect the mirror of the tip after cleaning. (vi) Dry the mirror carefully with a paper towel. (vii) Check to make sure it is free of lint, stains, and other kinds of dirt.	
	(a) Wrap the tip using a self-adhesive pouch or heat-sealed pouch.	
	(b) Sterilization using a steam autoclave class B (EN13060) and cycles at 121/134°C with drying.	
	(c) Storage in proper condition.	

Disposable plastic sleeve [21]		(i) Dispose of scanner sleeves according to standard operating procedures or local regulations for the disposal of contaminated medical waste.

*Type of disinfectant* [19]	(i) For optical windows and scanner tips: denatured alcohol (ethyl alcohol or ethanol) – typically 60-70% Alc/Vol. (ii) Mixture free of impurities that can stain the mirror.	(i) Many commercial products. (ii) Follow the disinfectant manufacturers' instructions for appropriate contact time. (iii) Remove residual liquid disinfectant with a lint-free, clean cloth.

*Wipe* [19, 20]	A soft lint-free nonabrasive cloth.	(i) Disinfectant wipes.

*Prohibition on mirror tip of the use of* [19]	(i) Ammonia-based or chloride based solutions or acetone on any surface. (ii) Acetone or any oxidizing solutions to clean the tip mirror.	

*Disposal of scanner tip*	Normally as other clinical waste.	Normally as other clinical waste.

**Table 3:** Some advices for better scanning.

Target	Actions
To avoid lint, stains, and dirt on the optical components:	Select disinfectants that do not produce faded stains and are nontoxic [4, 22].
	Do not allow any solution to dry.
	Sterilization in wrapped pouches to protect the optical parts and to guarantee the use of sterile tip.
	Put outside the pouch a type 5 chemical integrators (UNI EN ISO 11140), to avoid the possible interference of their released products.
	Attention before and during steam sterilization: in particular, it is important:
	Check water quality, the cleanliness of the steam autoclave camera and trays, autoclave loading, and perfect drying of the wrapped pouches.

To protect optical component from damage:	Put the pouch far from other devices.
	Use absorbent TNT gauze for protection.
	It is not known if it is better: (a) to put the mirror tip towards the paper or the plastic side of the barriers, (b) up or down in the autoclave camera.

To prevent fast deterioration of the plastic parts of the unit:	Use single-use wipes soaked with disinfectant, which also act quickly against antibiotic-resistant strains and have good compatibility with optical and plastic parts [4, 22].

## Abbreviations

ADA: American Dental Association
DHCP: Dental Healthcare Personnel
DISR: Dental Implant Supported Restoration
DL: Dental Laboratory
DT: Dental Technician
CAD/CAM: Computer-Aided Design/Computer-Aided Manufacturing
CCT: CAD/CAM Technology
CDC: Centres for Disease Control and Prevention
HBV: Hepatitis B Virus
FDA: Food and Drug Administration
HIV: Human Immunodeficiency Virus
MD: Medical Device
MRSA: * Methicillin-Resistant Staphylococcus Aureus*
PMMA: Poly(methyl methacrylate)
MSDS: Material Safety Data Sheet
PPE: Personal Protective Equipment.
